# Impact of probiotic *Saccharomyces boulardii* on the gut microbiome composition in HIV-treated patients: A double-blind, randomised, placebo-controlled trial

**DOI:** 10.1371/journal.pone.0173802

**Published:** 2017-04-07

**Authors:** Judit Villar-García, Robert Güerri-Fernández, Andrés Moya, Alicia González, Juan J. Hernández, Elisabet Lerma, Ana Guelar, Luisa Sorli, Juan P. Horcajada, Alejandro Artacho, Giuseppe D´Auria, Hernando Knobel

**Affiliations:** 1 Department of Infectious Diseases, Hospital del Mar, Barcelona, Spain; 2 IMIM (Hospital del Mar Medical Research Institute, Institut Hospital del Mar d'Investigacions Mediques), Barcelona, Spain; 3 Department of Medicine, Universitat Autònoma de Barcelona, Spain; 4 Joint Unit of Research in Genomics and Health, Foundation for the Promotion of Health and Biomedical Research in the Valencian Community (FISABIO) and Cavanilles Institute of Biodiversity and Evolutionary Biology (Universitat de València), València, Spain; 5 CIBER en Epidemiología y Salud Pública, Madrid, Spain; 6 Reference Laboratory of Catalonia, Barcelona, Spain; Rush University, UNITED STATES

## Abstract

Dysbalance in gut microbiota has been linked to increased microbial translocation, leading to chronic inflammation in HIV-patients, even under effective HAART. Moreover, microbial translocation is associated with insufficient reconstitution of CD4+T cells, and contributes to the pathogenesis of immunologic non-response. In a double-blind, randomised, placebo-controlled trial, we recently showed that, compared to placebo, 12 weeks treatment with probiotic *Saccharomyces boulardii* significantly reduced plasma levels of bacterial translocation (*Lipopolysaccharide-binding protein* or LBP) and systemic inflammation (IL-6) in 44 HIV virologically suppressed patients, half of whom (n = 22) had immunologic non-response to antiretroviral therapy (<270 CD4+Tcells/μL despite long-term suppressed viral load). The aim of the present study was to investigate if this beneficial effect of the probiotic *Saccharomyces boulardii* is due to modified gut microbiome composition, with a decrease of some species associated with higher systemic levels of microbial translocation and inflammation. In this study, we used 16S rDNA gene amplification and parallel sequencing to analyze the probiotic impact on the composition of the gut microbiome (faecal samples) in these 44 patients randomized to receive oral supplementation with probiotic or placebo for 12 weeks. Compared to the placebo group, in individuals treated with probiotic we observed lower concentrations of some gut species, such as those of the C*lostridiaceae* family, which were correlated with systemic levels of bacterial translocation and inflammation markers. In a sub-study of these patients, we observed significantly higher parameters of microbial translocation (LBP, soluble CD14) and systemic inflammation in immunologic non-responders than in immunologic responders, which was correlated with a relative abundance of specific gut bacterial groups (*Lachnospiraceae* genus and Proteobacteria). Thus, in this work, we propose a new therapeutic strategy using the probiotic yeast *S*. *boulardii* to modify gut microbiome composition. Identifying pro-inflammatory species in the gut microbiome could also be a useful new marker of poor immune response and a new therapeutic target.

## Introduction

Recent studies have shown that gut microbiota is impaired in HIV-patients, even after effective Highly Active Antirretroviral Therapy (HAART), and a large number of disease-associated bacteria have been identified. HIV-infection severely damages the gastrointestinal mucosal barrier resulting in microbial translocation [1–2], which in turn leads to continuous systemic inflammation and disease progression despite effective HAART [3–6]. Using high-resolution profiling of the bacterial community by 16S rDNA gene amplification and pyrosequencing, previous studies have identified a dysbiotic gut pattern in HIV-infected individuals, characterized by increased microbial translocation, chronic inflammation and hyperactivation of CD4+T cells, despite achieving long-term virologic suppression [7–11]. Moreover, microbial translocation is associated with insufficient reconstitution of CD4+T cells, and contributes to the pathogenesis of immunologic non-response [12–16]. A recent study have reported that HIV gut microbiome must be controlled for HIV risk factors, and after stratifying for sexual orientation, there was no solid evidence of an HIV-specific dysbiosis, but HIV-1 infection remained consistently associated with reduced bacterial richness and the lowest gut bacterial richness was observed in immunologic non-responders patients [17].

Recently the intestinal microbiome has been proposed as a novel therapeutic target for reducing chronic inflammation [18–19] and various interventions such as pre-probiotics have been proposed to improve the resident gut microbiome [20–22]. Since the specific effects of HIV infection on the particular gut bacterial taxa that contributes to chronic immunoactivation are unclear, it seems reasonable to propose treatments to improve the gut bacterial richness and HIV associated immune dysfunction. However, the beneficial effects of probiotics are strain-dependent and not all interventions are equally effective [23–25]. *Saccharomyces boulardii* is a probiotic whose clinical efficacy, anti-inflammatory and immunomodulatory effects are supported by extensive previous studies [26–30]. We recently demonstrated that treatment with *S*. *boulardii* significantly decreased plasma levels of microbial translocation (*Lipopolysaccharide-binding protein* or LBP) and inflammation parameters such as cytokine IL-6 in 44 HIV-treated patients, half of whom had an immunodiscordant response to antiretroviral therapy [31]. The aim of the present study was to investigate if this beneficial effect of the probiotic *Saccharomyces boulardii* is due to a modification in the gut microbiome composition of this patients, with a decrease in some species associated with higher systemic levels of microbial translocation and inflammation. In this study, we used 16S rDNA tagging to analyze the gut microbiome communities in these 44 patients, to assess the impact of probiotic treatment with *S*. *boulardii* compared to placebo.

In a sub-study of these patients, we explore if the immunological non-response was associated with higher systemic inflammation and microbial translocation parameters and a distinct gut microbiome pattern.

## Subjects, material and methods

### Study design

**Fig 1:**
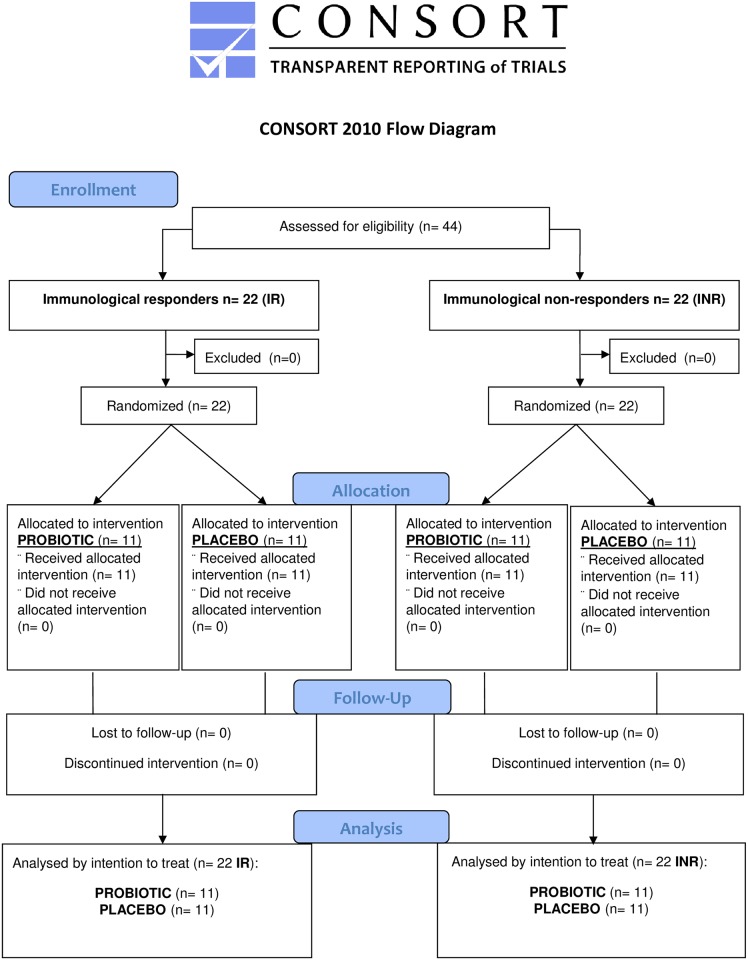
CONSORT flow diagram.

We performed a double-blind randomized, placebo-controlled trial to analyze the composition of the gut microbiota, microbial translocation, and inflammation parameters in 44 chronic HIV-infected patients with undetectable plasma viral load (<20 copies/mL) for at least 2 years and a stable HAART regimen. Half of the patients (n = 22) had already been previously selected as being immunodiscordant or immunological non-responders (INR), defined as individuals with an persistent unfavorable immunologic response (<270 CD4+Tcells/μL despite long-term suppressed viral load), while the other 22 had already been previously selected as immunologic responders (IR: CD4+T count >400 cells/μL). Once the patients had been included in the IR or INR group, they were double-blind randomized to oral supplementation with probiotic *S*. *boulardii* (capsules with 56.5 mg living yeasts, 2 capsules 3 times per day, n = 22), or placebo (2 capsules 3 times per day, n = 22) for 12 weeks. All 44 participants completed the study; none discontinued their participation because of side effects of the treatments or adverse events. (Fig 1. CONSORT Flow diagram). Markers of microbial translocation and inflammation, immunological and clinical data were collected before and after the intervention (Baseline and week 12), and follow-up visits were conducted until 48 weeks (See “S1 and S2 Protocols” Files). We recently reported the plasma findings in these patients [31]. All patients also provided 50gr of stool samples before and after the intervention, which were stored at -80°C within the first 24–48 hours; to allow us to compare gut microbiome composition before and after a 12-week treatment with probiotic or placebo. A computer-generated randomization list was used. Randomization was performed by a hospital pharmacist unrelated to the care of the patients, using sequentially numbered containers. The participants and the care providers were blinded after assignment to interventions.

The study was approved by the Clinical Research Ethics Committee of Hospital del Mar Medical Research Institute, number 2011/4508, on data 01/Feb/2012, according to the principles of the Declaration of Helsinki. Written informed consent was obtained from all participants before enrollment in the study. Patients were recruited between August 2012-July 2013. The study was registered during the enrollment because it had already been previously notified, registered and approved by the Hospital del Mar Medical Research Institute Ethics Committee. The authors confirm that related trial for this intervention is registered at ClinicalTrials.gov (PROB-VIH, Trial number: NCT01908049). “S1 Checklist”

### Microbial translocation markers

Serum levels of soluble CD14 were quantified using a ELISA kit (Quantikine^™^, R&D Systems, Minneapolis, MN) on a Quanta-Lyser^™^ 160 robotic workstation (Inova Diagnostics, San Diego, CA). Serum LBP was measured using the Immulite^™^ chemiluminescent immunometric assay on its automated analyzer (Immulite One, Siemens Healthcare^®^, Llanberis, UK).

### Inflammation markers

Milliplex MAP^™^ was used to analyze IL-6. Hs-CRP and β2 microglobulin were measured using the Immulite One^™^, and plasma fibrinogen with HemosIL^™^ reagents.

### Microbiome taxonomy analyses & bioinformatics

DNA was extracted from frozen stool samples and amplified by PCR targeting 16S gene regions using primers described elsewhere [32]. We performed library preparation followed by Illumina sequencing according to standard protocols described previously [33]. The taxonomic composition of the intestinal microbiota was characterized by grouping sequences into OTUs (Organizational Taxonomic Units). We used the QIIME open source algorithm for handling and interpretation of the data from the raw sequences; following quality assessment of sequence data, we assigned taxonomic affiliations using the RDP_classifier from the Ribosomal Database Project [34].

### Statistical analysis

No previous data were available to estimate the sample size; thus, for a Type I error rate of 0.05 and a 1-beta risk of 0.20 in a bilateral contrast, we computed that 22 patients were needed per study group to detect statistically significant differences between proportions. The assumed proportions for the power analysis for each group was 0.5. Continuous variables were expressed as the median and interquartile range, and discrete variables as percentages. Categorical variables were described as proportions. The Mann-Whitney U-test was used to compare medians, ANOVA to assess differences in continuous variables, and the Pearson's χ^2^ test to evaluate the association between categorical variables. We also conducted multivariate logistic regression analysis to study the correlation between microbial translocation and systemic inflammation parameters, and conditional backward stepwise regression analysis at baseline to identify parameters that were correlated with the immunodiscordance. The enter criteria was a p value<0.1. All the variables studied were selected based on the statistical significance. For the analysis of qualitative differences between responders and non-responders, we performed a median recoding of LBP, soluble CD4, β2 microglobuline and fibrinogen; the values obtained were defined as high or low. Statistical analyses were performed using SPSS software version 20.0 (SPSS, Inc., Chicago, IL) and several open source libraries in R (R Core Team 2014. R: A language and environment for statistical computing. R Foundation for Statistical Computing, Vienna, Austria. URL http://www.R-project.org/). The glm (R) function has been used in the logistic regression analysis.

## Results

### Patient characteristics

This study included 44 patients. Half of each group were immunological non-responders (INR), the median age was 47.5 years, 84% were male, and the risk factors for acquiring HIV infection were Heterosexual (HTX) (46%), Men who Have Sex with Men (MHSM) (38%) and Injection drug users (IDU) (16%). The profiles of demographic, biological and clinical parameters were similar in the probiotic and placebo groups [31] (“S1 Table”). All patients had suppressed viral load for a median of 4.7 years, with NNRTI-based (75%) or PI-based HAART (25%). The median nadir CD4+T was 108 cells/μl, and 41% had AIDS diagnosis.

### Microbial translocation is correlated with systemic inflammation

We tested for association between microbial translocation measurements and baseline parameters from 44 patients, and observed a statistically significant correlation between LBP levels and plasma levels of soluble CD14 (r = 0.48, p = 0.001), Hs-CRP (r = 0.63, p = 0.0001), β2 microglobulin (r = 0.59, p = 0.0001), Erythrocyte Sedimentation Rate-ESR (r = 0.57, p = 0.0001), Fibrinogen (r = 0.64, p = 0.0001), Cd4 nadir (r = -0.53, p = 0.0001), and IL-6 (r = 0.58, p = 0.0001). Using multivariate logistic regression analysis, we found that LBP was independently correlated with Hs-CRP, ESR and soluble CD14 (“S2 Table”).

### *Saccharomyces boulardii* produces changes in some gut bacterial communities

**Fig 2:**
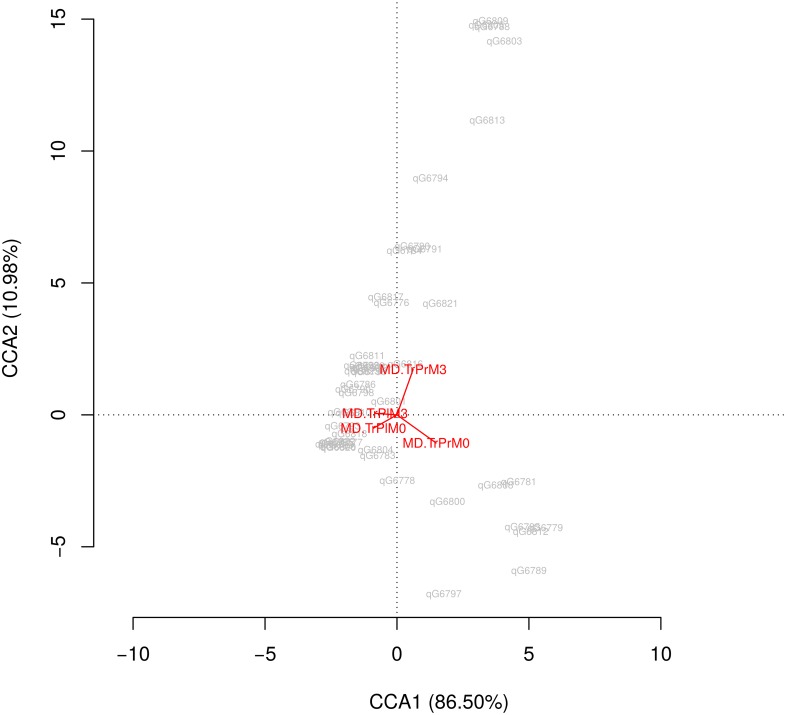
Canonical correspondence analysis.

**Fig 3:**
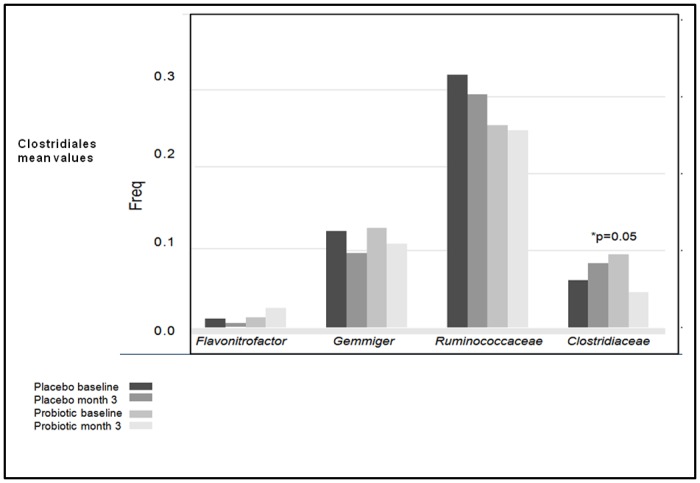
Changes in the proportion of Clostridiales after the interventions.

Canonical correspondance analysis shows the high influence of probiotic effect versus the time effect (vertical axis) (Fig 2). Changes in gut microbiome after the interventions are showed at “S1 Fig” (INRs group). After 12 weeks of probiotic treatment, we observed a significant decrease in levels of some Clostridiales species with respect to baseline compared with placebo (Fig 3). We also observed a significant decreased in *Catenibacterium* communities (2.65 to 0.04, p = 0.00003), and an increase in levels of Megamonas (p = 0.02) and Desulfovibrionales (Proteobacteria) (p = 0.05). These probiotic effects on gut microbiota composition were similar in the INR and IR groups.

**Fig 4:**
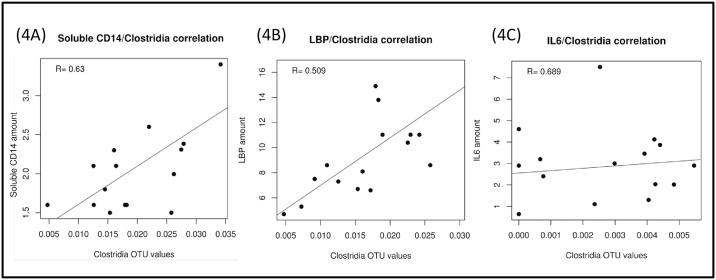
Correlations between relative abundance of Clostridiales and soluble CD14 (4A), LBP (4B) and IL6 (4C) before the intervention (INR). LBP, Lipopolisaccharide Binding-Proteine; IL6, interleukin-6.

**Fig 5:**
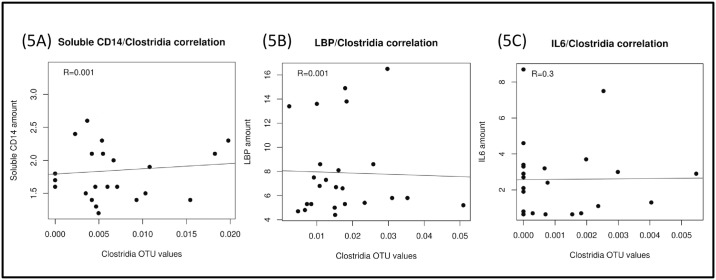
Correlations between relative abundance of Clostridiales and soluble CD14 (5A), LBP (5B) and IL6 (5C) after probiotic treatment. LBP, Lipopolisaccharide Binding-Proteine; IL6, interleukin-6.

We then investigated the correlation between these different stool bacterial populations, parameters of microbial translocation and systemic inflammation, and observed a statistically significant correlation between the proportion of Clostridia genera and plasma concentrations of soluble CD14 (r = 0.63, p = 0.03), LBP (r = 0.71, p = 0.009), and IL-6 (r = 0.69, p = 0.0008; Fig 4). These correlations were present at baseline in INR group but not in the IRs probably because IRs have a lower relative abundance of Clostridiaceae at baseline. The correlations are not statistically significant in probiotic treated group, probably because of a decrease in these bacterial communities due to the probiotic effect (Fig 5).

### Immunological non-response is associated with higher microbial translocation and the presence of some pro-inflammatory species

The baseline differences between the INR and IR groups are summarized in Table 1. Quantitative analysis of the baseline data indicates that the following variables showed statistically significant differences between the INR and IR group: age, current CD4+T count, CD4+T nadir, HCV coinfection, LBP and β2 microglobulin levels. The INR groups tended to have higher fibrinogen and soluble CD14 levels. Compared to the IR group, the INR group had a greater proportion of individuals with values greater than the median of LBP (68.2% *vs* 27.3%, p = 0.007); soluble CD14 (72.7% *vs* 40.9%, p = 0.03); β2 microglobuline (66.7% *vs* 33%, p = 0.022) and fibrinogen (65% *vs* 31.8%, p = 0.03).

**Table 1:** Differences between Immunological Responders (IR) and Non-Responders (INR).

	Responders (n = 22)	Non–Responders (n = 22)	*p-value*
**Demographics**			
Age (years) [SD]	44 (37–49)	52 (47–57)	0.014
Male [n (%)]	21 (95.5)	16 (72.7)	0.042
**HIV infection**			
Risk factor [n (%)]			
IDU	0 (0)	6 (100)	0.03
MHSM	12 (70.6)	5 (29.4)	0.06
HTX	10 (50)	10 (50)	1
Time with viral load <50 copies/ml (years) [median (IQR)]	4.5 (3–9.25)	5 (3.75–10)	0.463
*Nadir* CD4 cell count (/ml) [median (IQR)]	244 (105–293)	47.5 (9–113.75)	0.000
*Zenith* viral load (log10) [median (IQR)]	4.88 (4.58–5.38)	5.06 (4.39–5.41)	0.770
HCV co-infection [n (%)]	0 (0)	8 (100)	0.002
Current ART [n (%)]			
NNRTI	22 (68.8%)	10 (31.3%)	0.000
PI	0	11 (52.4%)	0.000
Absolute CD4T-count (cells/μl) [median (IQR)]	483 (413–630)	219 (169–266)	0.000
Absolute CD8T-count (cells/μl) [median (IQR)]	699.5(472.5–860.5)	594 (353–781)	0.199
**Microbial translocation parameters** [median (IQR)]			
LBP *(pg/mL)*	5.65 (5.2–6.5)	7.4 (6.1–8.7)	0.011
Soluble CD14 (μg/mL)	1.43 (1.31–1.93)	1.69 (1.55–2.11)	0.082
**Inflammation parameters**[median (IQR)]			
Hs-CRP(*mg/dl)*	0.22 (0.07–0.35)	0.16 (0.07–0.40)	0.874
IL -6*(pg/mL)*	1.85 (0.7–2.85)	2.7 (1.02–40.15)	0.466
Ig E (kU/L)	48.9 (16.8–157)	115 (21.6–360)	0.467
Fibrinogen (mg/dl)	257.5 (219–279)	293.5(259.5–324)	0.062
TNF-α*(pg/mL)*	10.9 (8.7–12.3)	12.7 (7.8–16.9)	0.190
ESR (mm/h)	7 (2–10.5)	10 (6–16.5)	0.100
Vit D 25 OH (ng/mL)	30.97(20.07–0.33)	37.04 (24.6–42.48)	0.253
β2microglobuline (μg/mL)	1.7 (1.54–2.06)	2.12 (1.93–2.86)	0.002
**Gut microbiome composition** (Relative abundance [%])			
*Lachnospiraceae* (Clostridia)	0.017%	0.093%	0.03
Proteobacteria	0.053%	0.248%	0.06

ART, antiretroviral therapy; PI, protease inhibitor; NNRTI, non-nucleoside; reverse transcriptase inhibitor; LBP, *Lipopolysaccharide-binding protein*; sCD14, soluble CD14; hs-CRP, high sensitivity C-reactive protein; IL-6, Interleukin 6; ESR, Erythrocyte Sedimentation Rate; IQR, interquartile range.

High LBP was associated with being an INR (Relative Risk = 5.71; 95% CI = 1.56–20.93; p = 0.015) and in the conditional backward stepwise regression analysis, high LBP was the only variable that could explain immunological non-response (OR = 6.22, 95% CI = 1.63–23.75, p = 0.007) (“S3 Table”).

Analyzing gut microbiome composition at baseline, we observed a higher proportion of some Firmicutes species (Clostridia) in the INR group than in the IR group. The proportion of Proteobacteria was higher in immunodiscordant patients; the proportion of *Lachnospiraceae* genera was significantly higher in the INR group (OR = 3.4, p = 0.05) (“S4 Table”).

## Discussion

This is the first clinical trial to use 16S rDNA sequencing to analyze changes in gut microbiome composition following treatment with *Saccharomyces boulardii*, and how these changes are correlated with microbial translocation and inflammation in HIV patients.

Microbial imbalance in the gut has been associated with increased microbial translocation, leading to chronic inflammation. In line with this, various microbial communities resident in the gut are correlated with plasma bacterial translocation markers (soluble CD14 and LBP) and pro-inflammatory cytokine interleukin-6 [10–11,35]. We recently reported a significant reduction in LBP and IL-6 levels in a small cohort of HAART-treated HIV patients supplemented with 12 weeks treatment with probiotic *S*. *boulardii* compared to placebo [31]. Here, we take a step further and use 16S rDNA tagging and high throughput sequencing to analyze microbiome profiles before and after the intervention, and to evaluate whether some predominant species are associated with increased bacterial translocation, systemic inflammation and poor immune response in these patients.

Following probiotic treatment, we observed a significant decrease in some Clostridiales, such as *Clostridiaceae* and *Catenibacterium* communities. Previous studies have used PCR or FISH techniques, rather than 16S rDNA sequencing, to evaluate the effects of supplementation with specific prebiotics in HAART-naïve infected HIV adults [21] and supplementation with probiotic *S*.*boulardii* in HIV-negative adults with enteritis [36]; these studies reported a consequent reduction in concentrations of pathogenic clostridia-related species, which are considered to be pro-inflammatory mucotropic bacteria, although these authors did not evaluate if this was correlated with a decrease in systemic inflammation. Recent studies have reported a higher proportion of *Catenibacterium* in HIV patients than in healthy individuals [9,11]. *Catenibacterium* is a Gram-positive, non-spore-forming and anaerobic genus from the family of Erysipelotrichidae. Taxa in this family were the most enriched [7], or a significant enrichment was reported [8–9], in the untreated HIV-infected subjects compared to HIV-uninfected individuals and was correlated with markers of microbial translocation and systemic inflammation in HIV patients [8]. In our study, the concentration of *Catenibacterium*, which have also been associated with other chronic diseases [37–39], also decreased following probiotic treatment.

We observed a relationship between bacterial translocation and various parameters of systemic inflammation. These data are consistent with previous studies demonstrating, in both treated and untreated HIV patients, a direct correlation between systemic parameters of bacterial translocation, and chronic immune activation and disease progression [40–48]. Furthermore, we found significant correlation between some bacterial taxa (Clostridia) and parameters of microbial translocation and systemic inflammation in immunological non-responders group (INR). Recent studies also have demonstrated a statistically significant correlation between some species of Clostridia, including *Lachnospiraceae*, and increased systemic immune activation parameters (TNF) in HIV-infected patients [11]. While the immunologic driving factors of gut dysbiosis related to HIV infection are likely to be complex interestingly, our study showed a significant reduction in these bacterial communities following probiotic treatment.

HIV-patients with deficient CD4+Tcell count recovery despite HAART have been defined as Immunological non-responders (INRs), and are estimated to account for up to 30% of all treated HIV-infected subjects. This is relevant because this subpopulation is associated with higher morbi-mortality. This phenomenon has been widely studied using different approaches, and its pathogenesis is known to be related to older age, nadir CD4 T-cell count, reduced thymic function, co-infection with hepatotropic viruses, and multiple genetic variants [12,13]. However, the ultimate cause of the discordant response is excessive CD4 T-cell destruction due to CD4+T hyperactivation [14], residual viral replication, and persistent antigenic stimulation due to microbial translocation. HAART-treated individuals have been reported to show an inverse correlation between levels of microbial translocation and sustained failure on CD4+T cell reconstitution [15,16]. A history of more advanced immunosuppression and the resulting damage to gut immunity and integrity are thought to be an important factor in the differences observed between groups in gut microbiome and markers of bacterial translocation and inflammation [49–51]. Our results are consistent with these findings, and extend them by comparing the composition of the gut microbial community in immunological responders and non-responders. Interestingly, the relative abundance of specific gut bacterial groups in INR patients is associated with greater bacterial translocation. Recent studies have shown that Proteobacteria, which are over-represented in our group of INRs, are responsible for much of the translocation after SIV-infection [52]; although the mechanisms remain unknown, Brenchley *et al*.[53] suggested their motility, high metabolic rate and possession of immune evasion genes as posibble reasons. Identifying gut species that contribute to the pathogenesis of immunologic non-response and persistent immune activation could be a new diagnostic and therapeutic tool in this group of HIV-patients with an increased risk of clinical progression and death.

The multiple anti-inflammatory mechanisms produced by *S*. *boulardii* provide molecular explanations to support its effectiveness in intestinal inflammatory states [30]. It has also been demonstrated that *S*. *boulardii* increases absolute numbers of the main habitual fermenting bacterial groups, and decreases concentrations of mucotropic occasional bacterial communities [54]. Several mechanisms of action have been identified directed against the host and pathogenic microorganisms, suppressing “bacteria overgrowth” and host cell adherence [30]. One limitation of this study is that, since *S*. *boulardii* is a yeast, we did not perform 16S rDNA gene amplification and parallel sequencing to demonstrate colonization by the probiotic, or changes in the gut mycobiome. A decrease in some dysbiotic bacterial genera, replacement of inflammogenic yeast and/or improved functional biostructure of colonic microbiota following *S*. *boulardii* treatment may prevent microbial translocation [54], although our study was not designed to determine the mechanism through which changes in gut microbiome composition alter its functional anatomy, metabolomics or mycobiome. Our study has some of the limitations of other studies in this topic. All are small works, and more extensive studies with better tools than 16s sequencing are needed before drawing definitive conclusions, including selection of the patients who could best respond to the probiotic treatment, as well as the doses and duration of the therapy. However, given the increasingly widespread use of metabolomic and metagenomic techniques, analysis of gut bacterial contents could be used in the future as a marker of a dysbiotic microbiome that contributes to chronic systemic inflammation and HIV progression. More importantly, the gut microbiome can be modified using certain strains of probiotics to generate a less pro-inflammatory profile; this new therapeutic target is worthy of further exploration.

## Conclusions

We observed a change in gut microbiome composition following probiotic treatment (*S*. *boulardii*), with a decrease in some species which are directly correlated with systemic levels of microbial translocation and inflammation. The use of specific probiotics could be a new therapeutic strategy for HIV patients. In addition, our data suggest that identifying pro-inflammatory species in the gut microbiome could be a new marker of poor immune response.

## Supporting information

S1 Protocol (DOC)

S2 Protocol (DOC)

S1 Checklist (DOC)

S1 Fig Changes in gut microbiome after the interventions (INRs group). INR, immunologic non-responders.
(TIF)

S1 Table Baseline characteristics of the patients. ART, antiretroviral therapy; PI, protease inhibitor; NNRTI, nonnucleoside; reverse transcriptase inhibitor; LBP, Lipopolisaccharide Binding-Proteine; sCD14, soluble CD14; hs-CRP, high sensitivity C-reactive protein; ESR, Erythrocyte Sedimentation Rate; IQR, interquartile range.
(DOCX)

S2 Table LBP Multivariate logistic regression analysis. LBP, Lipopolisaccharide Binding-Proteine; sCD14, soluble CD14, hs-CRP, high sensitivity C-reactive protein.
(DOCX)

S3 Table Backward stepwise logistic regression analysis. LBP_basal, Lipopolisaccharide Binding-Proteine at baseline; sCD14, soluble CD14; β2microglob, Beta 2 microglobuline.
(DOCX)

S4 Table INR logistic regression results. (CSV)
