# Supplementary material for: Impact of probiotic Saccharomyces boulardii on the gut microbiome composition in HIV-treated patients: A double-blind, randomised, placebo-controlled trial
Source: PLoS One. 2017 Apr 7;12(4):e0173802. doi: 10.1371/journal.pone.0173802 (PMC5384743; doi:10.1371/journal.pone.0173802)
Supplement: S1 Protocol — (DOC) [file pone.0173802.s001.doc]

**INDEPENDENT CLINICAL RESEARCH PROJECT: PROTOCOL**

|  | File nº |
| --- | --- |
|  | **SPI/2885/2011** |
| **TITLE: Treatment with probiotics (*Saccharomyces boulardii*) and its role in bacterial translocation and immune reconstitution in HIV infection** | |
|  | |
| Principal Investigator: **Judit Villar García** | |
| SUMMARY (Objectives and methodology):  **Objectives:** MAIN: To evaluate the parameters of bacterial translocation after treatment with probiotics (*Saccharomyces boulardii*) in HIV+ patients and its role on immune reconstitution. SECONDARY OBJECTIVES: 1) To analize the progress of immune activity markers after the administration of probiotics. 2) To determine the improvement of CD4+ lymphocytes and HIV viral load in patients after taking probiotics.  **Methods:** Design: A prospective randomized open controlled doble-blinded trial, to be performed at a tertiary care hospital in Barcelona. Subjects: Cronic HIV infected patients. Sample size: 44 cases. They´ll be divided in 2 groups: (1) Patients with CD4 +> 400 cells / ml and undetectable viral load for more than two years (22 cases) and (2) Patients with inmunodiscordancy, defined as patients with CD4 + T cells lower than 350 cells / ml despite 4-7 years of effective antiretroviral therapy. (22 cases). Intervention: Patients were randomized in 2 subgroups: (A) they´ll receive daily oral supplementation with *S. boulardii* for 3 months and (B) they ´ll receive placebo. Variables: bacterian lipopolisacarid levels (LBP), parameters of immune activation in plasma (sCD14, IFN-Υ, TNF, IL-2, IL-5, IL-6, IL-12) and fecal microbiome prior to the use of probiotics (baseline), at 3 and 6 months. Immunological and clinical data. Outcome measures: quantification of bacterial translocation levels, markers of activity and 16S rDNA gene amplification and parallel sequencing of fecal microbiome. Analysis: Comparison of variables before and after the intervention. The analysis will be performed by biological and immunological effectiveness. | |

**INDEPENDENT CLINICAL RESEARCH PROJECT: PROTOCOL**

|  | File nº |
| --- | --- |
|  | **SPI/2885/2011** |
| **PI: Judit Villar García** | |
| **Hypothesis**      Oral supplementation with probiotic *Saccharomyces boulardii* decreases enteropathy associated with HIV infection, and consequently, minimizes microbial translocation and chronic immune activation. | |
| **Objectives:**  Principal:  To evaluate the changes on bacterial translocation, systemic inflammation and gut microbiome after treatment with probiotic *(Saccharomyces boulardii)* compared with placebo in HIV-positive patients under an effective ART and its role on immune reconstitution.  Secondary objectives:  1) To analyze the changes in immunoactivity markers after administration of probiotics and their relationship with bacterial translocation.  2) To explore if the immunological non-response was associated with higher systemic inflammation and microbial translocation parameters and a different gut microbiome pattern. | |

| **Methods**: Population and Study Design  A single-center, randomized, double-blind, placebo controlled pilot study will be conducted in nonconsecutive HIV-1–infected patients aged 18 years or older.  Patients will be recruited at a tertiary care hospital (Institut Hospital del Mar d'Investigacions Mediques) in Barcelona, Spain.  All patients have chronic HIV infection, undetectable plasma viral load (,20 copies per milliliter) for at least 2 years, and a stable HAART regimen. It will be divided into 2 groups:  - Group 1 (n=22): Patients with CD4 +> 400 cells / ml and undetectable viral load for more than two years.  - Group 2(n=22): Discordant patients, deﬁned by an unfavorable persistent immunologic response (T-CD4+ cells <350 cells per microliter) despite long-term suppressed viral load (4-7 years).    Randomization:  A computer-generated randomization list will be used. Randomization will be performed by a hospital pharmacist unrelated to the care of the patients, using sequentially numbered containers. The participants and the care providers will be blinded after assignment to interventions.  Patients in both groups (1 and 2) will be randomized in 2 subgroups:  (A) 22 will receive daily oral *S. boulardii* supplementation for 3 months.  (B) 22 will receive white placebo patients (lactose) for 3 months.  Exclusion criteria:  - Patients who receive or have received in the last 6 months food supplements containing probiotics.  - Patients who have received antibiotic treatment in the last two months, or HIV patients who usually require to take antibiotics more than 2 times at year.  - Patients with presupposing poor adherence to dietary supplements or HAART.  - Changes in HAART regimen in the previous 2 years, and presence of a serious concomitant disease or substantial abnormalities in the laboratory tests that prevented study participation.  - Patients who do not sign the informed consent.  * Current antibiotic treatment during the study or the presence of any acute concomitant illness will not exclude patients out of the study.  **Intervention:**  - The intervention will be carried out an expert physician in Infectious Diseases.  - Daily treatment to all patients (S. boulardii VS placebo) will be prescribed at baseline in doses of 2 capsules every 12 hours, at least half an hour before intake, during 12 weeks.  - Extraction of blood samples and stool collection will be performed before starting probiotics (baseline) at 3 and 6 months.   In each case the following variables will be included in the data collection:  - Demographic affiliation, height, weight, toxic habits, depositional habit (BRISTOL scale).  - The HANDS scale of anxiety / depression will be apply.  - Dates of start and end of past and current antiretroviral medication will be recorded and concomitant medication administered during the study, including antibiotics.  - Any possible adverse effects of the intervention or serious adverse event will be recorded.  - Adherence to treatment will be registered, counting returned capsules on each visit.  **Study/Clinical Assessment:**  The primary end point of the study will be changes in serum LBP levels as a marker of microbial translocation.  Secondary endpoints will be as follows:   - Changes in markers of immune activity, including serum concentrations of soluble CD14 (sCD14), tumor necrosis factor alpha (TNF-a), interleukin 6 (IL-6), high-sensitivity C-reactive protein (Hs-CRP), B2 microglobulin, erythrocyte sedimentation rate, IL-2, IL-5, IL-12, D-dimer, IFN-Υ, ﬁbrinogen, Ig G, and Ig E. - Changes in immune status (T-CD4+ and T-CD8+ lymphocyte count) and HIV-viral load. - Analysis of gut microbioma by 16s rRNA sequencing. - Safety and tolerability.   **After screening for eligibility**, subjects will be visited at baseline (start of treatment), at week 12 (treatment discontinuation), and at week 24 (3 months after discontinuation of treatment).  Clinical and demographic data of the study patients will be collected from the medical records.  **At baseline**, eligible patients were randomized (1:1) to receive treatment with S. boulardii (2 capsules 3 times a day or 6 · 107 living bacteria) or placebo (2 capsules 3 times a day) for 12 weeks.  - Data collection: The data collected will be included in a database specifically designed for this protocol.  - Procedure:   - At each visit (Baseline, week 12 and week 24), clinical assessment and adverse events will be recorded, and blood samples were collected into 9 mL Vacuette serum separator tubes (Greiner Bio-One GmbH, Kremsmünster, Austria). Within 30 minutes after puncture, blood was centrifuged at 3000 rpm for 15 minutes, and serum samples were aliquoted and stored at 280°C until analysis. For all the tests, thawed serum samples were vortexed and centrifuged at 14,000 rpm for 10 minutes before testing. - All patients also provided 50gr of stool samples at each visit (Baseline, week 12 and week 24) , which will be stored at -80°C within the first 24-48 hours; to compare gut microbiome composition before and after a 12-week treatment with probiotic or placebo. - Once all samples will be collected, they will be sent together on dry ice to the Reference Laboratory of Catalonia, where they will be analyzed.   - Plasma and faecal sample Analysis:   - Inﬂammation Markers For analysis of IL-6 and TNF-a, the Milliplex MAP assay on a ﬂuorescent microsphere-based instrument (Merck Millipore, Merck KGaA, Darmstadt, Germany; Luminex/Bioplex200-Luminex Corp., Austin, TX and BioRad, Hercules, CA) will be used. Multianalyte proﬁling of serum concentrations will be performed using a commercially available multiplex immunoassay kit (HSCYTMAG-60K; Millipore, Billerica, MA). - Hs-CRP and b2 microglobulin will be measured using the Immulite chemiluminescent immunometric assay on its automated analyzer (Immulite One; Siemens Healthcare, Llanberis, United Kingdom). Plasma ﬁbrinogen will be measured using the Clauss coagulative method with HemosIL reactives (Instrumentation Laboratory, Bedford, MA). - Microbial Translocation Markers Serum levels of soluble CD14 will be quantiﬁed in duplicate with a commercially available enzyme-linked immunosorbent assay kit (Quantikine; R&D Systems, Minneapolis, MN) using a Quanta-Lyser 160 robotic workstation (Inova Diagnostics, San Diego, CA). - Serum LBP will be measured using the Immulite chemiluminescent immunometric assay on its automated analyzer. All assays will be performed according to the manufacturer’s instructions. - Fecal Microbiome Analyses: DNA will be extracted from frozen stool samples and amplified by PCR targeting 16S gene regions using primers described elsewhere ([Klindworth A](http://www.ncbi.nlm.nih.gov/pubmed/?term=Klindworth A%5BAuthor%5D&cauthor=true&cauthor_uid=22933715),[Nucleic Acids Res.](http://www.ncbi.nlm.nih.gov/pubmed/22933715) 2013). We will performed library preparation followed by Illumina sequencing according to standard protocols described previously (Caporaso JG, [ISME J.](http://www.ncbi.nlm.nih.gov/pubmed/?term=Caporaso+JG%2C+Lauber+CL%2C+Walters+WA%2C+Berg-Lyons+D%2C+Huntley+J%2C+Fierer+N%2C+Owens+SM%2C+Betley+J%2C+Fraser+L%2C+Bauer+M%2C+Gormley+N%2C+Gilbert+JA%2C+Smith+G%2C+Knight+R.+2012.+Ultra-high-throughput+microbial+community+analysis+on+the+Illumina+HiSeq+and+MiSeq+platforms.+2012.+ISME+J) 2012). The taxonomic composition of the intestinal microbiota will be characterized by grouping sequences into OTUs (Organizational Taxonomic Units).   Statistical analysis and bioinformatics:   - Student t test was used to assess differences between 2 mean values, and a Mann–Whitney U test was performed when data were not normally distributed. Either the x2 test or the Fisher exact test was used to test the degree of association of categorical variables. Statistical signiﬁcance was set at P , 0.05. Statistical analyses were performed using SPSS software version 20.0 (SPSS, Inc, Chicago, IL). - Microbiome Taxonomy Analyses & Bioinformatics: We used the QIIME open source algorithm for handling and interpretation of the data from the raw sequences; following quality assessment of sequence data, we assigned taxonomic affiliations using the RDP_classifier from the Ribosomal Database Project ( Wang, Q, Appl Environ Microbiol. 2007).   Ethics Committee  The study was approved by the Ethics Committee of Hospital del Mar Medical Research Institute, according to the principles expressed in the [Declaration of Helsinki](http://www.wma.net/en/30publications/10policies/b3/index.html) (annexed). Written informed consent will be obtained from all participants before enrollment in the study (annexed).  **Intervention:**  Aspects related to the investigational medicinal product (manufacture, labeling, distribution ...)  Probiotic: Trade name: Ultra - Levura ® lyophilized   - Responsible for manufacturing: BIOCODEX. - Drug marketed and distributed in Spain by Zambon Laboratories S.A. - Presentation: container with 50 capsules. - quantitative composition: Each capsule contains 1,000 million live bacteria (107) *Saccharomyces boulardii 17* lyophilised: 56.5 mg. Excipients: Sucrose (92.3mg), magnesium stearate q.s. - Laboratory Sales Price: 5.51 € / bottle of 50 tablets.   White Placebo: Sucrose   - Responsible for manufacturing: Kern Pharma   **Funding sources:** Helps independent clinical research of the Ministry of Health No. SPI / 2885/2011 |
| --- |
|  |

| **INDEPENDENT CLINICAL RESEARCH PROJECT: PROTOCOL** | File nº |
| --- | --- |
|  | **SPI/2885/2011** |
| **PI: Judit Villar García** | |
| **Workplan**  The project will take place at the Hospital del Mar, following different stages:  PHASE 1: (Duration: 3 months)  - Selection of patients included in the study.  - Check circuit extraction and processing of samples.  PHASE 2: (6 months)  - Recruitment, inclusion and randomization of patients.  - Administration of probiotics in a controlled manner Basal visit at 3 months and 6 months in the randomly selected group.  - Obtain baseline data and plasma samples and feces, at 3 and 6 months of patients.  - Storing data in the database designed for this purpose.  - Preliminary analysis of the data.  PHASE 3: (12 months)  - Monitoring patients in the last month.  - Final data analysis.  - Publication of the results and communicate them in an international congress. | |

| **ANNEX: INFORMATION SHEET FOR THE PATIENT AND INFORMED CONSENT** | |  |
| --- | --- | --- |
| PACIENT INFORMATION **Study title: Treatment with probiotics (Saccharomyces boulardii) and its role in bacterial translocation and immune reconstitution in HIV infection. SPI / 2885/2011**  **Hospital**  Departmento de Medicina Interna- Enfermedades Infecciosas  Hospital del Mar  Paseo Marítmo, 25-29  08003 Barcelona, Spain  Name and surname of patient  -------------------------------------------------- -------------------------------------------------- ----------  1. NATURE AND PURPOSE OF THE STUDY  This is an informed consent document. It contains a full explanation of the study to which we invite you to participate and a consent form that will ask you to sign if you choose to participate. It is being asked to participate because you have a chronic HIV infection. There is a need to advance knowledge about the use of probiotics in clinical practice. For this reason, the Hospital del Mar is conducting a prospective controlled study with nutritional supplements to gather additional information about its effects on health, particularly on the immune system. It is given for this purpose, Saccharomyces boulardii (Ultra-levura®), which must be taken daily following the pre-registrations from your doctor. The methods for this study and present FCI have also been examined and approved by the Ethics Committee for Clinical Research of this hospital and the Spanish Agency for Medicines and Health Products (AEMPS).  2. ESTIMATED TIME OF STUDY AND expected number of participants  This study will involve approximately 44 people. You will be in the studio for a maximum period of 6 months.  3. EXPLANATION OF PROCEDURES  Once you have met the requirements to be part of the study, a physician will interview and examine your medical records to obtain the following information:  • Date of birth, gender and race / ethnicity.  • Height, weight, and general medical history, including toxic habits, depositional habit and mood. dates of start and end of past and current medication were recorded.  • Laboratory results of his latest blood test and stool sample.  You will be assigned a random group, with 50% chance of being assigned to each group, depending on the assignment will take the probiotic or placebo for 3 months. Allocation to one or other group can not be chosen by you or your doctor. Neither you nor your doctor will know if you are taking probiotics or placebo, although they can know at any time if necessary.    Analysis will be performed on the first visit, at 3 and 6 months. On each visit it must provide a stool sample of less than 24 hours ago and keep frozen until deliver. During the study, you can not take other dietary supplements, including dairy products containing probiotics, as this could alter the results.  FOLLOW UP  health information and treatment will be collected for at least 6 months. This information will come from regular visits to your doctor and your medical history and any test being performed as part of their standard treatment.  4. NOTICE OF SIDE EFFECTS  - No contraindications or side effects described by taking Saccharomyces boulardii.  - Nor they have described interactions with drugs you are taking for HIV.  - Must notify your doctor as soon as possible if you receive or been given antibiotic treatment, since it could alter the results of the study.  - You must report any adverse event to experience your doctor. If you experience a serious adverse event such as an illness requiring hospitalization, report it to your study doctor immediately or as soon as possible.  - In case of pregnancy (if you are a woman) during their participation in this study, you should inform your doctor, but may continue taking the treatment.  5. RIGHT TO WITHDRAW THE STUDY  Participation in this study is voluntary. You can decide not to participate or you can leave the study at any time. If you decide not to participate or withdraw from the study, this will have no penalty for you. You will not lose any benefits he was entitled nor his decision will affect access to health care in the future. To withdraw from the study, data and samples have been taken and will be analyzed as planned, unless you state that does not allow the time to abandon the study.  6. CONFIDENTIALITY  It will be need the access to your medical history, including your past medical records and test results for the purpose of this study. By signing the informed consent you authorize this access and, if necessary, the study staff will contact with other health professionals. The access to your personal information will be limited to doctor study and his collaborators, health authorities and the Ethics Committee for Clinical Research. They all act in accordance with the professional secrecy inherent to their activity, if necessary, to check the data and study procedures, but always maintaining confidentiality in accordance with the current legislation.  If you want to withdraw from the study, you should notify your study doctor. This may try to contact you if you leave the studio without telling. If you decide to leave the study, the study staff will not collect more information about you, although information about you that has already been collected may continue to be used as specified above. If you have any questions, we recommend that you discuss them wiyh the doctor in charge of this study.   Compliance with current legislation about the confidentiality of health information  The data processing will follow the principles established in the Law 14/2007 on Biomedical Research in Law 15/1999 on Protection of Personal Data and Royal Decree 1720/2007 approving the Regulation implementing approved of Law 15/1999 on Protection of Personal data.  7. ECONOMIC COMPENSATION  The doctor or institution shall not receive any compensation from the company that markets the probiotic. This study will have no cost to you, as it will be given the probiotic for free during visits. You will not receive economic compensation for participating in the study.  8. RESPONSE TO QUESTIONS ABOUT THIS STUDY  Before signing this form, you can ask about anything you do not understand. The study staff will answer your questions before, during and after the study.  Contact with Dr. ____________, telephone ____________ for any of the following reasons:  • If you have any questions about your participation in this study.  • If you have questions, concerns or complaints about the research.  **INFORMED CONSENT**  **Study title: Treatment with probiotics (Saccharomyces boulardii) and its role in bacterial translocation and immune reconstitution in HIV infection. SPI / 2885/2011**  I agree to participate in this prospective, randomized, placebo versus a probiotic as specified in the information form that has given me.  I understand that my participation in this study is voluntary and I can leave the study at any time, without specifying the reason. I have read all the information on this study and all my questions have been answered satisfactorily. I agree that my data for this study are used as specified in the form and which may be transferred for evaluation, stored and managed electronically anonymously. I understand that I will receive a copy of this consent, which I signed, together with the information sheet which has given me.  I have been informed that my medical records may be audited by the ethical / scientific Hospital Committee or by the Ethics Committee for Clinical Research and health authorities, if appropriate, and give my consent for it .  Name:………………………….………………….…….  (Patient or legal representative)  Signature and Date…………………………………….  (Patient or legal representative)  I confirm that the patient has been informed about the nature, purpose and possible outcomes of this prospective, randomized and controlled study. The patient information has been discussed and provided with the informed consent form.  Name: …………………………………….  (Responsible for explaining the consent)  Signature and Date ……………………………………….. |  | |
|  | | |
