# Supplementary material for: Impact of probiotic Saccharomyces boulardii on the gut microbiome composition in HIV-treated patients: A double-blind, randomised, placebo-controlled trial
Source: PLoS One. 2017 Apr 7;12(4):e0173802. doi: 10.1371/journal.pone.0173802 (PMC5384743; doi:10.1371/journal.pone.0173802)
Supplement: S2 Protocol — (DOC) [file pone.0173802.s002.doc]

**PROYECTO DE INVESTIGACIÓN CLINICA INDEPENDIENTE: PROTOCOLO**

|  | | | | | Expediente nº | | |
| --- | --- | --- | --- | --- | --- | --- | --- |
|  | | | | | **SPI/2885/2011** | | |
| **TITULO**: **Tratamiento con probióticos (*Saccharomyces boulardii)***  **y su papel en la translocación bacteriana y la reconstitución inmune en la infección por el VIH.** | | | | | | | |
| Solicitante: **Judit Villar García** | | | | | | | |
| Investigador/a principal: **Judit Villar García** | | | | | | | |
| TIPO DE PROYECTO: | INDIVIDUAL | X | MULTICENTRICO |  | |  | |
| NOMBRE COORDINADOR/A (MULTICENTRICOS DE VARIAS SOLICITUDES): | | | | | | | |
| RESUMEN (Objetivos y metodología del proyecto):  **Objetivos:**  PRINCIPAL: Evaluar la evolución de la translocación bacteriana tras el tratamiento con bífidus (*Saccharomyces boulardii)* en pacientes VIH+ y su papel sobre la reconstitución inmune. OBJETIVOS SECUNDARIOS: 1) Analizar la evolución de los marcadores de actividad inmune tras la administración de los bífidus. 2) Estudiar la evolución de los linfocitos CD4+ y la carga viral del VIH en los pacientes tras la toma de los probióticos.  **Metodología**: Diseño: Estudio controlado prospectivo aleatorizado doble ciego a realizar en un hospital de tercer nivel en Barcelona. Sujetos: Pacientes afectos de infección crónica por VIH. Tamaño muestral: 44 casos. Se dividirán en 2 grupos: (1) Pacientes con linfocitos CD4+>400 cél/ml y carga viral indetectable desde hace más de 2 años (22 casos) y (2) Pacientes con criterios de inmunodiscordancia, definida como recuperación de células TCD4+<350 células/ml a pesar de 4–7 años de tratamiento antirretroviral efectivo (22 casos). Intervención: Los pacientes se randomizarán en 2 subgrupos: (A) recibirán suplementación oral diaria con *S. boulardii* durante 3 meses y (B), recibirán placebo durante 3 meses. Variables a estudio: niveles de lipopolisacáridos bacterianos en plasma (LBP) y parámetros de activación inmune (sCD14, IFN-Υ, TNF, IL-2, IL-5, IL-6, IL-12)**,** y análisis de microbioma fecal previo al uso de probióticos (basal) a los 3 y 6 meses. Se determinarán asimismo datos inmunológicos y clínicos. Medidas de resultado: cuantificación de niveles de translocación bacteriana, marcadores de actividad y análisis del microbioma fecal mediante secuenciación 16s rDNA. Análisis de datos: Se compararán variables a estudio antes y después de la intervención. El análisis se realizará por eficacia biológica e inmunológica. | | | | | | |  |
| **TITLE: Treatment with probiotics (*Saccharomyces boulardii*) and its role in bacterial translocation and immune reconstitution in HIV infection** | | | | | | | |
| SUMMARY (Objectives and methodology):  **Objectives:** MAIN: To evaluate the parameters of bacterial translocation after treatment with probiotics (*Saccharomyces boulardii*) in HIV+ patients and its role on immune reconstitution. SECONDARY OBJECTIVES: 1) To analize the progress of immune activity markers after the administration of probiotics. 2) To determine the improvement of CD4+ lymphocytes and HIV viral load in patients after taking probiotics.  **Methods:** Design: A prospective randomized open controlled doble-blinded trial, to be performed at a tertiary care hospital in Barcelona. Subjects: Cronic HIV infected patients. Sample size: 44 cases. They´ll be divided in 2 groups: (1) Patients with CD4 +> 400 cells / ml and undetectable viral load for more than two years (22 cases) and (2) Patients with inmunodiscordancy, defined as patients with CD4 + T cells lower than 350 cells / ml despite 4-7 years of effective antiretroviral therapy. (22 cases). Intervention: Patients were randomized in 2 subgroups: (A) they´ll receive daily oral supplementation with *S. boulardii* for 3 months and (B) they ´ll receive placebo. Variables: bacterian lipopolisacarid levels (LBP), parameters of immune activation in plasma (sCD14, IFN-Υ, TNF, IL-2, IL-5, IL-6, IL-12) and fecal microbiome prior to the use of probiotics (baseline), at 3 and 6 months. Immunological and clinical data. Outcome measures: quantification of bacterial translocation levels, markers of activity and 16S rDNA gene amplification and parallel sequencing of fecal microbiome. Analysis: Comparison of variables before and after the intervention. The analysis will be performed by biological and immunological effectiveness. | | | | | | | |

**PROYECTO DE INVESTIGACIÓN CLINICA INDEPENDIENTE: PROTOCOLO**

|  | Expediente nº |
| --- | --- |
|  | **SPI/2885/2011** |
| **IP: Judit Villar García** | |
| **Hipótesis de trabajo**  La suplementación alimentaria oral con *Saccharomyces boulardii* disminuye la enteropatía asociada a la infección por VIH, y, como consecuencia, minimiza las translocación microbiana y la inmunoactivación crónica secundaria. | |
| **Objetivos**  Principal:  Evaluar la evolución de la translocación bacteriana y los cambios en el microbioma intestinal tras el tratamiento con bífidus (*Saccharomyces boulardii)* en pacientes VIH positivos con TAR efectivo y su papel sobre la reconstitución inmune.  Objetivos secundarios:  1) Analizar la evolución de los marcadores de actividad inmune tras la administración de los probióticos y su relación con la translocación bacteriana y la inflamación sistémica.  2) Estudiar si los pacientes inmunodiscordantes presentan mayor translocación bacteriana, inflamación sistémica y un patrón diferente de microbioma intestinal. | |

| **Metodología**: Diseño del estudio  Estudio experimental controlado, prospectivo aleatorizado, doble ciego.  Ámbito: Institut Hospital del Mar d'Investigacions Mediques. Laboratorio de Referencia de Catalunya.  Período de estudio: Enero - diciembre del 2012  Población en estudio. Criterios de inclusión/exclusión  La población en estudio está constituida por pacientes con infección crónica por VIH controlados en el Hospital del Mar.  Se dividirán en 2 grupos (22 pacientes por grupo):  - Grupo 1: Pacientes con linfocitos CD4+ > 400 cél/ml y carga viral indetectable desde hace más de dos años.  - Grupo 2: Pacientes con criterios de inmunodiscordancia, definida como recuperación de células T CD4+ inferior a 350 células/ml a pesar de 4–7 años de tratamiento antirretroviral efectivo (carga viral indetectable).  Aleatorización:  Se utilizará una lista de aleatorización generada por computadora, que llevara a cabo un farmacéutico del hospital no relacionados con el cuidado de los pacientes, los recipientes serán numerados secuencialmente.  Tanto los participantes como los investigadores desconocerán el contenido de la asignación de las intervenciones (doble ciego).  Los pacientes de ambos grupos se randomizarán en 2 subgrupos:  (A) pacientes que recibirán suplementación oral diaria con *S. boulardii* durante 3 meses, tras los cuales pararán el tratamiento.  (B) pacientes que recibirán placebo blanco (lactosa)durante 3 meses.  Criterios de exclusión:   - Pacientes que reciban o hayan recibido en los últimos 6 meses complementos alimenticios que contengan probióticos. - Pacientes que hayan recibido tratamiento antibiótico en los últimos 2 meses, o que, por su patología de base diferente al VIH, requieran la toma de antibióticos más de 2 veces al año. - La toma de antibióticos durante el estudio, no excluirá a los pacientes del mismo. - Pacientes a los que se les presuponga mala adherencia a los suplementos alimenticios. - Pacientes que hayan cambiado el TAR en los últimos 3 meses. - Pacientes que no firmen el consentimiento informado.   Intervención:  - La intervención será llevada a cabo un médico experto en Enfermedades Infecciosas (IP).  - Se prescribirán a todos los pacientes tratamiento diario (*S. boulardii* VS placebo) en dosis de 2 cápsulas cada 12 horas, como mínimo media hora antes de la ingesta.  - Se realizará extracción de muestras sanguíneas y recogida de heces antes de iniciar los probióticos (basal) a los 3 y 6 meses.  - La realización del estudio no conllevará ninguna modificación de la práctica clínica habitual salvo la obtención de muestras sanguíneas con la periodicidad señalada y la suplementación alimentaria durante los meses que se hayan determinado de forma aleatoria.  Variables principales y secundarias  En cada caso se incluirán las siguientes variables en la hoja de recogida de datos:   - Datos de filiación y demográficos, altura, peso, hábitos tóxicos, hábito deposicional. - Se aplicará la escala HANDS de ansiedad/depresión. - Se registrarán las fechas de inicio y de finalización de la medicación antirretroviral pasada y actual, así como de la medicación concomitante administrada durante el estudio, incluída la toma de antibióticos. - La toma de antibióticos durante el estudio, no excluirá a los pacientes del mismo, ya que la cepa a estudio (*S. boulardii 17*) es resistente a antibióticos, pero se registrará como una variable por si pudiera alterar los resultados. - Se registrarán posibles efectos adversos derivados de la toma de bífidus. - Se registrará la adherencia al tratamiento, con conteo de las cápsulas que se devuelven en cada visita.   **Variables a estudio:**  Se realizará extracción analítica y recogida de heces a todos los pacientes previo al uso de probióticos (basal), a los 3 y 6 meses, determinándose los siguientes parámetros:  Variable principal:  Niveles de lipopolisacáridos bacterianos en plasma: Lipopolisaccharide Binding Proteine (LBP), como marcador de translocación bacteriana.  Variables secundarias:   - Marcadores de actividad inmune: CD14 soluble (sCD14), Interferón gamma (IFN-Υ), Factor de necrosis tumoral (TNFa), IL-2, IL-5, IL-6, IL-12, VSG, PCR ultrasensible, D-dímero, fibrinógeno, Ig G, e Ig E.   - Parámetros que determinan el estado inmunológico: cifra de linfocitos CD4 +, CD 8 +.  - Carga viral del VIH.  - Análisis de microbioma intestinal mediante secuenciación 16s rDNA.  - Recogida de datos: Los datos recogidos se incluirán en una base de datos específicamente diseñada.  - Procedimiento:   - Los análisis se realiza´ran en muestras de sangre extraídas en Hospital de Día de Enfermedades Infecciosas. Se usarán tubos de 9 mL Vacuette (Greiner Bio-One GmbH, Kremsmünster, Austria). A los 30 minutes tras la punción, la sangre se centrifugará a 3000 rpm durante 15 minutes, y las muestras de suero serán alicuotadas y congeladas a -80°C hasta su análisis posterior. - Para todas las determinaciones, las muestras de suero descongeladas se someterán a vórtice y se centrifugarán a 14.000 rpm durante 10 minutos. Además de las alícuotas requeridas en la extracción analítica habitual para el control de la infección por VIH, se añadirá un tubo de suero de 3 mL y uno de EDTA. - Los pacientes aportarán 50gr de heces recogidas en un contenedor estéril hace <24 h y se congelarán inmediatamente, siendo conservadas una vez entreagadas al investigador a -80ºC. - Éste procedimiento se repetirá en las visitas BASAL, a los 3 y a los 6 meses, en ambas ramas del estudio. - Una vez recogidas todas las muestras del estudio, se enviarán en nieve carbónica al Laboratorio de Referencia de Cataluña, donde serán analizadas.   -Análisis de las muestras:   - Para el análisis de marcadores de inflamación (IL-6 y FNT-a) se usará MAPA basado en microesferas fluorescentes (Merck Millipore, Merck KGaA, Darmstadt, Alemania; Luminex / Bioplex200-Luminex Corp., Austin, TX y BioRad, Hercules , CA). El perfil multianálisis de las concentraciones séricas se realizará usando un kit de inmunoensayo múltiplex disponible comercialmente (HSCYTMAG-60K; Millipore, Billerica, MA). - La PCR ultrasensible y la B2 microglobulina se medirán utilizando el ensayo inmunométrico quimioluminiscente Inmulite en su analizador automático (Inmulite Uno; Siemens Healthcare, Llanberis, Reino Unido). - El fibrinógeno plasmático se medirá utilizando el método de Clauss coagulativa con reactivos HemosIL (Instrumentation Laboratory, Bedford, MA). - La translocación microbiana (niveles de séricos de CD14 soluble) se cuantificará por duplicado con un kit de inmunoensayo multiplex comercial (Quantikine; R & D Systems, Minneapolis, MN) utilizando una estación de trabajo robotizada Quanta-Lyser 160 (Inova Diagnostics, San Diego, CA). La concentración más baja detectable sCD14 es 0,25 ng / ml . - La LBP sérica se midió usando el ensayo inmunométrico quimioluminiscente Immulite en su analizador automatizado. - Todos los ensayos se realizarán de acuerdo con las instrucciones del fabricante.Para el análisis de control de calidad se utilizarán los materiales que incorporan los kits, y será interno. - Análisis del microbioma fecal: Se extraerá ADN de las muestras de heces congeladas y se amplificarán por PCR las regiones 16S usando cebadores descritos previamente ([Klindworth A](http://www.ncbi.nlm.nih.gov/pubmed/?term=Klindworth A%5BAuthor%5D&cauthor=true&cauthor_uid=22933715),[Nucleic Acids Res.](http://www.ncbi.nlm.nih.gov/pubmed/22933715) 2013); Se realizará secuenciación Illumina de acuerdo a protocolos estándar descritos previemente (Caporaso JG, [ISME J.](http://www.ncbi.nlm.nih.gov/pubmed/?term=Caporaso+JG%2C+Lauber+CL%2C+Walters+WA%2C+Berg-Lyons+D%2C+Huntley+J%2C+Fierer+N%2C+Owens+SM%2C+Betley+J%2C+Fraser+L%2C+Bauer+M%2C+Gormley+N%2C+Gilbert+JA%2C+Smith+G%2C+Knight+R.+2012.+Ultra-high-throughput+microbial+community+analysis+on+the+Illumina+HiSeq+and+MiSeq+platforms.+2012.+ISME+J) 2012). La composición taxonómica de la microbiota intestinal se caracterizará mediante agrupación de secuencias en Otus (Unidades de Organización taxonómica).   Análisis de los datos:   - Se analizarán los datos mediante el paquete estadístico SPSS 19.0. El análisis se realizará de “eficacia biológica e inmunológica” y según el tratamiento recibido y su duración. Las variables cuantitativas se describirán con la media (y desviación estándar) o la mediana (y la amplitud intercuartil) en función de su homogeneidad, y las cualitativas en números absolutos y porcentajes. Las variables categóricas (porcentajes) se compararán mediante una prueba de X2 (o de Fisher, si procede) y las cuantitativas mediante una prueba de t de Student (o la prueba U de Mann-Whitney, en su caso). En caso de que hubiera diferencias significativas o relevantes entre las características basales de los grupos en comparación, respecto a alguna variable, se ajustará el análisis por dicha variable, mediante un análisis de regresión logística. Las incidencias y riesgos relativos se calcularán con un intervalo de confianza (IC) del 95%. Se considerará significativo un valor de p < 0.05. - Para el análisis taxonómico y bioinformático del microbioma fecal se utilizará el algoritmo de código abierto QIIME para el manejo e interpretación de los datos de las secuencias en bruto; después de la evaluación de calidad de los datos de secuencias, se asignarán afiliaciones taxonómicos utilizando el RDP_classifier del Proyecto de base de datos ribosomal ( Wang, Q, Appl Environ Microbiol. 2007).   Aspectos éticos: Este estudio ha sido aprobado por el Comité de Ética del Hospital del Mar –Instituto de Investigaciones biomédicas según la Declaración de Helsinki (anexo). La firma del consentimiento informado será requisito indispensable para la inclusión de los pacientes en el estudio de manera izada (anexo).  Aspectos relacionados con el medicamento en investigación(fabricación, etiquetado, distribución…)   - Probiótico: Nombre comercial: **Ultra – Levura** ® liofilizada   Responsable de la fabricación: *BIOCODEX.*  Medicamento comercializado y distribuído en España por Laboratorios Zambon S.A.  Presentación: envase con 50 cápsulas.  Composición cuantitativa: Cada cápsula contiene 1.000 millones de gérmenes vivos ( 10^7^) ***Saccharomyces boulardii* 17** liofilizado: 56.5 mg. Excipientes: Sacarosa (92.3mg), estearato magnésico c.s.p.  Precio de Venta del Laboratorio: 5,51 €/frasco de 50 comprimidos.   - Placebo blanco: Sacarosa   Responsable de la fabricación: *Kern Pharma*  Fuentes de financiación: Ayuda a la Investigación Clinica independiente del Ministerio de Sanidad nº **SPI/2885/2011** | |
| --- | --- |
|  | |
|  | Expediente nº |
|  | **SPI/2885/2011** |
| **IP: Judit Villar García** | |
| **Plan de trabajo**  El proyecto se realizará en el Hospital del Mar, siguiendo las diferentes **etapas**:  **FASE 1:** (Duración: 3 meses)   - Selección de los pacientes que se incluirán en el estudio. - Puesta a punto del circuito de extracción y procesamiento de las muestras.   **FASE 2**: (6 meses)   - Reclutamiento, inclusión y aleatorización de los pacientes. - Administración de probióticos de forma controlada en la visita Basal, a los 3 meses y a los 6 meses en el grupo seleccionado aleatoriamente. - Obtención de los datos basales y de las muestras en plasma y heces , a los 3 y a los 6 meses de los pacientes. - Almacenamiento de los datos en la base diseñada para tal fin. - Análisis preliminar de los datos.   **FASE 3**: (12 meses)   - Finalización del seguimiento de los pacientes incluidos en el último mes. - Análisis definitivo de los datos. - Publicación de los resultados y comunicación de los mismos en un congreso internacional. | |

| **ANEXOS: HOJA DE INFORMACIÓN PARA EL PACIENTE Y CONSENTIMIENTO INFORMADO** | |  |
| --- | --- | --- |
| INFORMACIÓN PARA EL PACIENTE **Título del estudio: Tratamiento con probióticos (*Saccharomyces boulardii)***  **y su papel en la translocación bacteriana y la reconstitución inmune en la infección por el VIH. SPI/2885/2011**  **Hospital o Institución**  Departmento de Medicina Interna- Enfermedades Infecciosas  Hospital del Mar  Paseo Marítmo, 25-29  08003 Barcelona, Spain  **Nombre y apellidos del paciente**  --------------------------------------------------------------------------------------------------------------   1. NATURALEZA Y OBJETIVO DEL ESTUDIO   Este es un documento de consentimiento informado. Contiene una explicación completa del estudio al que le invitamos a participar y un formulario de consentimiento que le pediremos que firme si decide participar. Se le está pidiendo que participe porque usted tiene una infección crónica por el VIH. Existe una necesidad de avanzar en los conocimientos sobre el uso de probióticos en la práctica clínica. Por este motivo, el Hospital del Mar está realizando un estudio prospectivo y controlado con suplementos alimenticios para recopilar información adicional sobre sus efectos sobre la salud, en concreto sobre su sistema inmunológico. Se le administrará, con éste fin, *Saccharomices boulardii* (Ultra-levura®), que deberá tomar diariamente siguiendo las preinscripciones de su médico. Los métodos para este estudio y el presente FCI también han sido examinados y aprobados por el Comité Ético de Investigación Clínica de este hospital y por la Agencia Española de Medicamentos y Productos Sanitarios (AEMPS).   1. DURACIÓN PREVISTA DEL ESTUDIO Y NÚMERO PREVISTO DE PARTICIPANTES   En este estudio participarán aproximadamente 44 personas. Usted estará en el estudio durante un periodo máximo de 6 meses.  3. EXPLICACIÓN DE LOS PROCEDIMIENTOS A SEGUIR  Una vez haya cumplido los requisitos para formar parte del estudio, un médico le entrevistará y estudiará su historia clínica para obtener la siguiente información:   - Fecha de nacimiento, sexo y raza/origen étnico. - Altura, peso, e historia clínica general, incluidos los hábitos tóxicos, hábito deposicional y estado anímico. Se registrarán las fechas de inicio y de finalización de la medicación pasada y actual. - Resultados de laboratorio de sus últimos análisis de sangre y la muestra de heces.   Se le asignará un grupo al azar, con un 50% de posibilidades de ser asignado a cada grupo, y dependiendo de la asignación tomará el probiótico o el placebo, durante 3 meses. Su asignación a uno u otro grupo no podrá ser elegida por usted ni por su médico. Ni usted ni su médico conocerán si está tomando probióticos o placebo, aunque se podrá saber en cualquier momento si fuera necesario.    Se le realizarán análisis en la primera visita, a los 3 y a los 6 meses. En cada visita deberá aportar una muestra de heces de hace menos de 24h y mantenerla congelada hasta entregarla. Durante el estudio, usted no podrá tomar otros suplementos alimenticios, incluídos lácteos, que contengan probióticos, ya que esto podría alterar los resultados.  SEGUIMIENTO  Se recogerá información sobre su salud y sobre el tratamiento durante un mínimo de 6 meses. Esta información procederá de las visitas habituales a su médico y de su historia clínica así como de cualquier prueba que se realice como parte de su tratamiento estándar.  4. NOTIFICACIÓN DE EFECTOS SECUNDARIOS   - No se han descrito contraindicaciones ni efectos secundarios al tomar *Saccharomyces boulardii.* - Tampoco se han descrito interacciones con los fármacos que usted esté tomando para el VIH. - Deberá notificar a su médico en cuanto le sea posible si va a recibir o le han administrado tratamiento antibiótico, ya que podría alterar los resultados del estudio. - Deberá notificar cualquier acontecimiento adverso que experimente a su médico. Si experimenta un acontecimiento adverso grave, como una enfermedad que requiera hospitalización, informe de ello a su médico del estudio inmediatamente o en cuanto le sea posible. - En caso de embarazo (si es usted una mujer) mientras dure su participación en este estudio, deberá informar a su médico, aunque podrá continuar tomando el tratamiento.   5. DERECHO A RETIRARSE DEL ESTUDIO  La participación en este estudio es voluntaria. Puede decidir no participar o puede abandonar el estudio en cualquier momento. Si decide no participar o abandonar el estudio, esto no tendrá ninguna penalización para usted. No perderá ningún beneficio al que tuviera derecho ni su decisión afectará a su acceso a la asistencia médica en el futuro. Al retirarse del estudio, los datos y las muestras que ya se hayan tomado se analizarán tal como está previsto, a menos que usted declare que no lo permite en el momento de abandonar el estudio.  6. CONFIDENCIALIDAD  **Acceso a su historia clínica:** Los médicos del Centro que participan en este estudio necesitarán acceso a su historia clínica, incluidos sus registros médicos pasados y los resultados de los análisis para la finalidad de este estudio. Con la firma del consentimiento informado usted autoriza este acceso y, en caso necesario, el personal del estudio podrá ponerse en contacto con su médico de familia y con otros profesionales sanitarios para obtener acceso a su El acceso a su información personal estará limitado al médico del estudio y a sus colaboradores, a las autoridades sanitarias y al Comité Ético de Investigación Clínica. Todos ellos actuarán de acuerdo con el secreto profesional inherente a su actividad, en caso necesario, para comprobar los datos y procedimientos del estudio, pero manteniendo siempre su confidencialidad de conformidad con la legislación actual.  **Abandono del estudio**  Si desea retirarse del estudio, deberá comunicárselo a su médico del estudio. Este podrá intentar contactar con usted si abandona el estudio sin comunicárselo, para saber si desea continuar. Si decide abandonar el estudio, el personal del estudio no recogerá más información sobre usted, aunque la información sobre usted que ya haya sido recogida podrá seguir utilizándose según lo especificado anteriormente. Si tiene alguna pregunta, le recomendamos que se la comente a su médico del estudio.  **Cumplimiento de la legislación actual sobre la confidencialidad de la información sobre su salud**  El tratamiento de los datos seguirá los principios establecidos en la Ley 14/2007 de Investigación Biomédica, en la Ley Orgánica 15/1999 de Protección de Datos de Carácter Personal y en el Real Decreto 1720/2007 por el que se aprueba el Reglamento de desarrollo de la Ley Orgánica 15/1999 de Protección de Datos de Carácter Personal.  7. COMPENSACIÓN  El médico o su institución no recibirán ninguna compensación por parte del laboratorio que comercializa el probiótico. Este estudio no tendrá ningún coste para usted, ya que se le administrará el probiótico de forma gratuita durante las visitas. No recibirá compensación económica por participar en el estudio.  8. RESPUESTA A LAS PREGUNTAS ACERCA DE ESTE ESTUDIO  Antes de que firme este formulario, puede preguntar sobre cualquier cosa que no entienda. El personal del estudio responderá a sus preguntas antes, durante y después del estudio.  Póngase en contacto con ____________ en ____________ por alguno de los siguientes motivos:   - Si tiene alguna pregunta sobre su participación en este estudio. - Si tiene preguntas, preocupaciones o quejas sobre la investigación.   Antes de que firme este formulario, puede preguntar sobre cualquier cosa que no entienda. El personal del estudio responderá a sus preguntas antes, durante y después del estudio.    **CONSENTIMIENTO INFORMADO**  **Título del estudio: Tratamiento con probióticos (*Saccharomyces boulardii)***  **y su papel en la translocación bacteriana y la reconstitución inmune en la infección por el VIH. SPI/2885/2011**  Acepto participar en éste estudio prospectivo, aleatorizado, con un probiótico versus placebo, tal como se especifica en el formulario informativo que se me ha entregado.  Entiendo que mi participación en este estudio es voluntaria y que puedo abandonar el estudio en cualquier momento, sin especificar el motivo. He leído toda la información sobre este estudio y todas mis preguntas han sido respondidas satisfactoriamente. Acepto que mis datos para el estudio arriba mencionado se utilicen tal como se especifica en el formulario y que podrán transferirse para su evaluación, guardarse y gestionarse de forma electrónica de manera anónima. Entiendo que recibiré una copia de este consentimiento, que he firmado, junto con la hoja de información que se me ha entregado.  Se me ha informado de que mi historia clínica podrá ser objeto de una auditoría por parte del Comité ético/científico del Hospital o por parte del Comité Ético de Investigación Clínica y las autoridades sanitarias, en caso de que proceda, y doy mi consentimiento para ello.  Nombre:………………………….………………….…….  (Paciente o representante legal)  Firma……………………………………….. Fecha…………………………………….  (Paciente o representante legal)  Confirmo que el paciente ha sido informado sobre la naturaleza, el objetivo y los posibles resultados de este estudio prospectivo, randomizado y controlado con un probiótico. La información para el paciente ha sido comentada y proporcionada junto con el formulario de consentimiento informado.  Nombre…………………………………………… Fecha…………………………………….  (Responsable de explicar el consentimiento)  Firma……………………………………….. |  | |
|  | | |
