# Supplementary figures and images for: Impact of probiotic Saccharomyces boulardii on the gut microbiome composition in HIV-treated patients: A double-blind, randomised, placebo-controlled trial
Source: PLoS One. 2017 Apr 7;12(4):e0173802. doi: 10.1371/journal.pone.0173802 (PMC5384743; doi:10.1371/journal.pone.0173802)

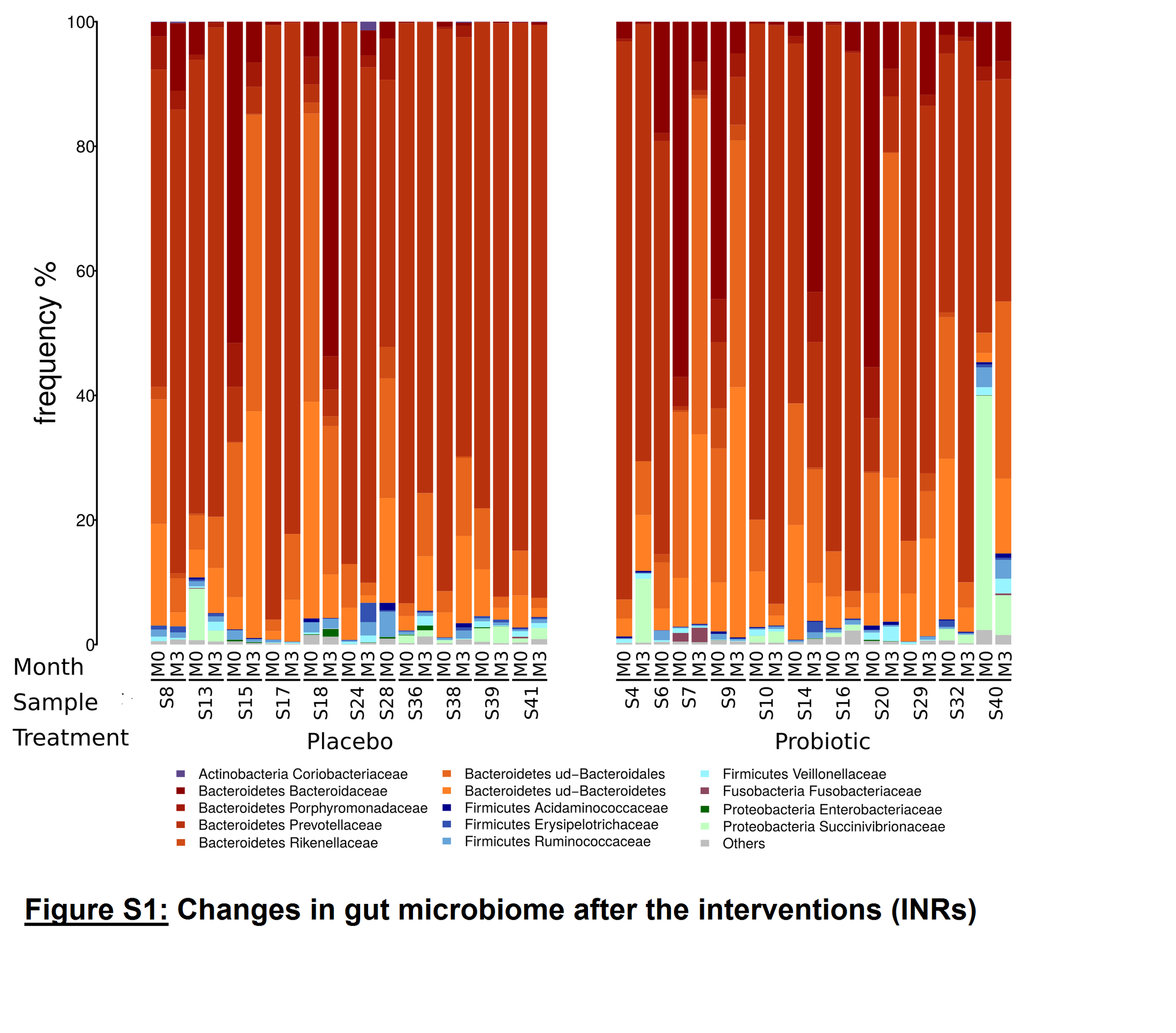

Supplement: S1 Fig — INR, immunologic non-responders. (TIF) [file pone.0173802.s004.tif]
