# Supplementary material for: Impact of probiotic Saccharomyces boulardii on the gut microbiome composition in HIV-treated patients: A double-blind, randomised, placebo-controlled trial
Source: PLoS One. 2017 Apr 7;12(4):e0173802. doi: 10.1371/journal.pone.0173802 (PMC5384743; doi:10.1371/journal.pone.0173802)
Supplement: S1 Table — ART, antiretroviral therapy; PI, protease inhibitor; NNRTI, nonnucleoside; reverse transcriptase inhibitor; LBP, Lipopolisaccharide Binding-Proteine; sCD14, soluble CD14; hs-CRP, high sensitivity C-reactive protein; ESR, Erythrocyte Sedimentation Rate; IQR, interquartile range. (DOCX) [file pone.0173802.s005.docx]

|  | Probiotic | Placebo | *p* |
| --- | --- | --- | --- |
| n | 22 | 22 | 1 |
| Demographics |  |  |  |
| Age (years) [SD] | 49.45 (7.75) | 45.5 (7.75) | 0.16 |
| Male [n (%)] | 20 (90.9) | 17 (77.3) | 0.41 |
| Ethnicity [n (%)] |  |  |  |
| White | 17 (77.3) | 17 (77.3) | 1 |
| HIV infection |  |  |  |
| Risk factor [n (%)] |  |  |  |
| IDU | 3 (13.6) | 4 (18,2) | 0.80 |
| MHSM | 9 (40.9) | 8 (36.4) | 0.80 |
| HTX | 10 (45.5) | 10 (45.5) | 1 |
| Time since HIV diagnosis (years) [median (IQR)] | 15 (6-21) | 10 (6-20) | 0.39 |
| Time with viral load <50 copies/m (years) [median (IQR)] | 5.5 (5-10) | 4 (3-9) | 0.06 |
| *Nadir*  CD4 cell count (/ml) [median (IQR)] | 90 (49-243) | 126 ( 33-268) | 0.93 |
| AIDS diagnosis [n (%)] | 10 (45.5) | 8 (36.4) | 0.54 |
| HIV RNA (% < 50 copies/ml) [median (IQR)] | 100 | 100 | 1 |
| Discordant [n (%)] | 11 (50%) | 11 (50%) | 1 |
| Z*enith*  viral load ( log10) [median (IQR)] | 4.93 (4-5.43) | 4.91 (4.67- 5.31) | 0.76 |
| VHC co-infection [n (%)] | 5 (22.7) | 3 (13.6) | 0.70 |
| Current ART [n (%)] |  |  |  |
| NNRTI | 17 (77.3) | 16 (72.7) | 1 |
| PI | 5 (22.7) | 6 (27.3) | 1 |
| Absolute CD4 T-count (cells/ µl) [median (IQR)] | 328 (220-457) | 328 (207-503) | 0.82 |
| CD4 T-cell count (% lymphocytes) [median(IQR)] | 24.1 ( 19.2-30.4) | 24.6 ( 16.2-32.2) | 0.92 |
| Absolute CD8 T-count (cells/ µl) | 697 (538-833) | 481.5 (350-791) | 0.17 |
| CD8 T-cell count (% lymphocytes) | 45.8 ( 41.4-53.45) | 37.9 (32-55.4) | 0.17 |
| Microbial translocation parametres [median (IQR)] |  |  |  |
| LBP *( pg/mL)* | 6.6 (5.8- 8.6) | 5.95 ( 5.3-8.3) | 0.35 |
| Soluble CD14 (µg/mL) | 1.7 (1.4-2.1) | 1.55 (1.3-1.9) | 0.37 |
| Immflamation parametres [median (IQR)] |  |  |  |
| hs-CRP (*mg/dl)* | 0.24 ( 0.07- 0.48) | 0.14 (0.07- 0.34) | 0.59 |
| IL -6 *(pg/mL)* | 2.7 (1.5- 3.3) | 1.4 ( 0.7-3.4) | 0.12 |
| Fibrinogen (mg/dl) | 265 (225.5- 346.5) | 273.5 (236-317) | 0.91 |
| TNF-α *(pg/mL)* | 11.9 (8.7-13.2) | 10.6 (8-16) | 0.68 |
| ESR (mm/h) | 10 (7-18) | 7 (5-10) | 0.11 |
| β2microglobuline (µg/mL) | 2.11 ( 1.87- 2.45) | 1.77 (1.51- 2.13) | 0.02 |
