# Supplementary material for: Impact of probiotic Saccharomyces boulardii on the gut microbiome composition in HIV-treated patients: A double-blind, randomised, placebo-controlled trial
Source: PLoS One. 2017 Apr 7;12(4):e0173802. doi: 10.1371/journal.pone.0173802 (PMC5384743; doi:10.1371/journal.pone.0173802)
Supplement: S2 Table — LBP, Lipopolisaccharide Binding-Proteine; sCD14, soluble CD14, hs-CRP, high sensitivity C-reactive protein. (DOCX) [file pone.0173802.s006.docx]

| sCD14 (µg/mL) | 2.15 (CI 95%: 0.18 - 4.12) | p : 0.03 |
| --- | --- | --- |
| CD4 nadir (cells/ µl) | -0.59 (CI 95%: -1.41 - 0.23) | p: 0.15 |
| IL-6 (pg/mL) | 0.11 (CI 95%: -0.01 - 0.21) | p: 0.06 |
| Fibrinogen (mg/dl) | -0.03 (CI 95%: -0.19 – 0.14) | p: 0.75 |
| β2microglobuline (µg/mL) | -1.09 (CI 95%: -2.62 - 0.45) | p: 0.16 |
| Erythrocyte Sedimentation Rate (ESR) (mm/h) | 1.28 (CI 95%: 0.31 - 2.26) | p: 0.01 |
| Hs-CRP (mg/dl) | 1.97 (CI 95%: 0.06 - 3.90) | p: 0.04 |
