# Supplementary material for: Impact of probiotic Saccharomyces boulardii on the gut microbiome composition in HIV-treated patients: A double-blind, randomised, placebo-controlled trial
Source: PLoS One. 2017 Apr 7;12(4):e0173802. doi: 10.1371/journal.pone.0173802 (PMC5384743; doi:10.1371/journal.pone.0173802)
Supplement: S3 Table — LBP_basal, Lipopolisaccharide Binding-Proteine at baseline; sCD14, soluble CD14; β2microglob, Beta 2 microglobuline. (DOCX) [file pone.0173802.s007.docx]

|  | | B | E.T. | Wald | gl | Sig. | Exp(B) | CI 95% for EXP(B) | |
| --- | --- | --- | --- | --- | --- | --- | --- | --- | --- |
|  |  |  |  |  |  |  |  | Lower | Upper |
| 1^st^ Step | LBP_Basal | 1,135 | ,782 | 2,103 | 1 | ,147 | 3,110 | ,671 | 14,417 |
|  | Gender | 1,374 | 1,251 | 1,207 | 1 | ,272 | 3,952 | ,340 | 45,883 |
|  | β2microglob | -,839 | ,802 | 1,093 | 1 | ,296 | ,432 | ,090 | 2,082 |
|  | Fibrinogen | -1,028 | ,753 | 1,864 | 1 | ,172 | ,358 | ,082 | 1,565 |
|  | sCD14 | -,237 | ,825 | ,082 | 1 | ,774 | ,789 | ,157 | 3,977 |
|  | Constant | ,273 | ,843 | ,105 | 1 | ,746 | 1,314 |  |  |
| 2^nd^ Step | LBP_Basal | 1,184 | ,765 | 2,395 | 1 | ,122 | 3,267 | ,730 | 14,627 |
|  | Gender | 1,409 | 1,245 | 1,280 | 1 | ,258 | 4,091 | ,356 | 46,970 |
|  | β2microglob | -,926 | ,744 | 1,550 | 1 | ,213 | ,396 | ,092 | 1,702 |
|  | Fibrinogen | -1,036 | ,753 | 1,892 | 1 | ,169 | ,355 | ,081 | 1,553 |
|  | Constant | ,188 | ,788 | ,057 | 1 | ,811 | 1,207 |  |  |
| 3^rd^ Step | LBP_Basal | 1,385 | ,735 | 3,551 | 1 | **,060** | 3,993 | ,946 | 16,857 |
|  | β2microglob | -,913 | ,728 | 1,574 | 1 | ,210 | ,401 | ,096 | 1,671 |
|  | Fibrinogen | -,913 | ,728 | 1,574 | 1 | ,210 | ,401 | ,096 | 1,671 |
|  | Constant | ,192 | ,782 | ,060 | 1 | ,806 | 1,212 |  |  |
| 4^th^ Step | LBP_Basal | 1,584 | ,710 | 4,977 | 1 | **,026** | 4,875 | 1,212 | 19,608 |
|  | Fibrinogen | -1,008 | ,710 | 2,016 | 1 | ,156 | ,365 | ,091 | 1,467 |
|  | Constant | -,341 | ,646 | ,278 | 1 | ,598 | ,711 |  |  |
| 5^th^ Step | LBP_Basal | 1,828 | ,684 | 7,152 | 1 | **,007** | 6,222 | 1,630 | 23,757 |
|  | Constant | -,981 | ,479 | 4,198 | 1 | ,040 | ,375 |  |  |
|  | | | | | | | | | |
